# Inequities in Health Service Needs Among Older Adults During COVID-19: A Gender-Stratified Analysis Using Longitudinal SHARE Data

**DOI:** 10.1177/00469580251371425

**Published:** 2025-09-09

**Authors:** Aija Bukova-Žideļūna, Anda Ķīvīte-Urtāne, Lauma Sprinģe, Ilze Priedīte, Diāna Baltmane, Šime Smolič

**Affiliations:** 1 Riga Stradins University, Latvia; 2 University of Zagreb, Croatia

**Keywords:** health service needs, older adults, SHARE study, pandemics, COVID-19

## Abstract

This study examined sociodemographic and health-related determinants of self-reported unmet health service needs to better understand the factors contributing to inequities among adults aged 50 and older during the COVID-19 pandemic in Latvia. Data from the longitudinal SHARE study—Corona Surveys 1 and 2, and Wave 8—were analysed using logistic regression. A total of 647 cases from Latvia (62.9% women) were included, with a mean age of 68.6 years (±9.6). During the pandemic, 29.5% of adults aged 50 and older encountered barriers to accessing healthcare services. Gender-stratified analysis revealed that older adults from urban areas, men with higher education, and women with chronic conditions and activity limitations were at significantly higher risk of experiencing unmet health service needs. The study reveals significant disparities in health service needs among older adults in Latvia during the COVID-19 pandemic, emphasising the need for targeted interventions to reduce inequalities and improve access for vulnerable populations in public health crises.

## Introduction

Older adults faced heightened barriers to healthcare during the COVID-19 pandemic, underscoring the need to identify system-level vulnerabilities and inequities. This study aims to investigate the sociodemographic and health-related determinants of self-reported unmet health service needs among individuals aged 50 and older in Latvia during the first year of the pandemic (March to June/July 2020) and 1 year later (June/July 2021). We hypothesise that unmet health service needs in this population were associated with sociodemographic factors and health-related characteristics.

Latvia confirmed its first COVID-19 case on March 3, 2020^1^ and declared a pandemic emergency from March 13 to June 9, 2020,^2^ during which significant restrictions were imposed on planned inpatient and outpatient healthcare services.^3^ An analysis of 14-day COVID-19 case rates and death rates indicated that Latvia had some of the lowest rates among European Union (EU) countries during what is commonly referred to as the first wave of the pandemic in the spring of 2020.^4^ However, a substantial decrease in the total number of consultations for patients with non-communicable diseases provided by general practitioners and specialists was observed.^5^ The second state of emergency was declared in Latvia from November 6, 2020, to April 6, 2021, with less strict public health measures. However, unlike the first months of the pandemic, Latvia did not manage to keep the number of COVID-19 cases below the EU average.^1^

### Disruptions in Healthcare

The ability of healthcare systems to effectively provide essential prevention and treatment services for chronic non-communicable diseases was compromised during pandemics. A study examining the COVID-19 pandemic found that 75% of the 163 member states of the World Health Organisation (WHO) either substantially reduced or entirely suspended healthcare services for patients with chronic non-communicable diseases. Among the countries included in the study, 65% reported maintaining essential scheduled outpatient services.^6^ A study focussing on the COVID-19 pandemic during the spring of 2020 in Latvia revealed that patients, particularly those with chronic conditions, exhibited fear of infection, leading them to avoid consultations with general practitioners and specialists, hospital visits, and emergency medical services.^5^ Furthermore, research suggests that the suspension of planned health services during the first wave of COVID-19, combined with the overburdening of the healthcare system, may have negatively impacted non-communicable disease mortality during the first year of the pandemic in Latvia.^7^

### Vulnerable Older Adults

Previous studies have shown that older individuals who frequently use healthcare services tend to have more severe illnesses compared to those who use them infrequently.^8^ Studies analysing older adults during pandemics have shown that they faced delays in diagnosis and treatment, significant reductions in physician consultations, specialist referrals, and hospital admissions, and those in poorer health were more likely to have unmet health service needs.^9,10^ Older patients with chronic non-communicable diseases, such as diabetes, cardiovascular diseases, chronic respiratory conditions, and cancer, were at a higher risk of contracting and succumbing to COVID-19, as well as experiencing adverse health outcomes related to their underlying conditions.^11^ Disparities in healthcare access disproportionately impacted the elderly in poor pre-pandemic health, particularly among the oldest age groups, due to the cumulative effects of economic and medical vulnerabilities.^12^ Moreover, adverse health consequences appeared to be more severe for older patients.^13^ This may widen socioeconomic health inequalities among the ageing population.

Even before the COVID-19 pandemic, Latvia faced significant challenges in population health, with half of those aged 65 and over having at least 1 chronic disease in 2019.^14^ Furthermore, the country had the lowest number of healthy life years at age 65 among all EU member states in 2020.^15^ In the study from 2020 that analysed population vulnerability to COVID-19 in Europe, Latvia was identified as one of the countries at high risk due to both the proportion of the population aged 70 years and above and the rate of years lived with disability.^16^ These demographic and health indicators underscore the need to examine how the COVID-19 pandemic affected healthcare access for older populations in Latvia. A thorough analysis can help develop evidence-based strategies to strengthen healthcare system resilience and ensure that vulnerable groups are not left behind but are systematically included in strategies to address future public health emergencies effectively.

## Methods

This study is reported in accordance with the STROBE (Strengthening the Reporting of Observational Studies in Epidemiology) guidelines.

### Study Design and Population

We used data from the Survey of Health, Ageing, and Retirement in Europe (SHARE) for our study.^17-19^ SHARE is a multidisciplinary, cross-national panel database of microdata on the health, socioeconomic status, and social and family networks of individuals aged 50 or older. We utilised the SHARE Corona Survey 1 dataset, released on 10 February 2022, with data collected via Computer-Assisted Telephone Interviews conducted between June and July 2020. Additionally, we used the SHARE Corona Survey 2 dataset, released on 10 December 2022, based on follow-up interviews conducted during the summer of 2021, one year after the first SHARE Corona Survey. Methodological details of the data collection process are described in Scherpenzeel et al.^20^ Furthermore, we incorporated data from the SHARE Wave 8 regular panel. As a longitudinal study, SHARE comprises both panel and regular refreshment samples. The exact sample size varies by wave and is designed to maximise the net sample size. Participants in Latvia are selected through household-level probability sampling with screening, based on a nationally representative sample. Stratification is applied to ensure population representativeness. For this study, the final sample includes individuals observed in all 3 surveys: the first and second SHARE Corona surveys and Wave 8 of the regular SHARE survey, comprising 647 respondents from Latvia.

### Unmet Health Service Needs

Our primary variable of interest was whether an individual experienced unmet health service needs during the first (2020) and/or second (2021) years of the pandemic in Latvia.

Individuals’ subjective unmet health service needs during the pandemic were assessed through the following survey questions in both Corona Surveys:

- “Since the outbreak of Corona, did you forgo medical treatment because you were afraid to become infected by the corona virus?”
- “Did you have a medical appointment scheduled, which the doctor or medical facility decided to postpone due to Corona?”
- “Did you ask for an appointment for a medical treatment since the outbreak of Corona and did not get one?”

One binary variable was constructed, equal to one, if the individual responded “yes” to at least one of these questions in any of the Corona Surveys. This variable captures unmet health service needs, defined as having forgone or postponed at least one medical appointment or treatment in general during both the first and second years of the pandemic, an approach similar to that described by Bergeot.^21^

### Sociodemographic Factors

Sociodemographic characteristics included gender (man/woman), age at interview (50-64/65 and older), education (primary/secondary/tertiary), partnership status (no partner/has a partner) and area lived (urban/rural). To evaluate economic activity before the pandemic, respondents were asked to indicate their current employment circumstances, which were categorised as either active or inactive. The financial situation was evaluated based on their reported ability to make ends meet since the onset of the pandemic and categorised as either good or poor.

### Health Status and Limitations

Health status was assessed using self-reported information on the number of chronic conditions (2 or fewer/ more than 2 chronic diseases). Functional limitations were evaluated using the Activities of Daily Living (ADL) Index and the Instrumental Activities of Daily Living (IADL) Index (no limitation/1 or more limitations). Participation restrictions were assessed using the Global Activity Limitation Indicator (GALI), based on the survey question: “For at least the past 6 months, to what extent have you been limited because of a health problem in activities people usually do?” (not limited/ limited). Methodological details of the indicators and modifications are described in Mehrbrodt et al.^22^

### Statistical Analysis

The prevalence of unmet health service needs was expressed as a percentage—the total 1—and stratified into sub-groups of independent variables. Descriptive statistics, including mean and standard deviation (SD), were calculated for continuous variables. Univariate and multivariate binary logistic regression analysis was performed to identify factors associated with the outcome variable (unmet health service needs). The multivariate model was constructed, adjusting for age, education, partnership status, area of residence, economic activity, and financial status. Results were considered statistically significant if *P* < .05. Data were processed using IBM SPSS Statistics (Statistical Package for the Social Sciences) version 26.0.

## Results

A total of 647 cases from Latvia (62.9% women) were included, with a mean age of 68.6 years (SD ± 9.6). About 29.5% of the individuals were unable to access some form of healthcare services during the pandemic in Latvia. Table 1 provides an overview of the distribution of unmet and no unmet service needs in the study population. After adjustment for gender, age, education, family status, area lived, economic activity and economic status, significantly higher odds of experiencing unmet health services needs were reported among women (odds ratio (OR = 1.6; 95% confidence interval [CI]: 1.04–2.4), those with secondary (OR = 1.8; 95% CI: 1.2–2.8) and tertiary education (OR = 2.1; 95% CI: 1.3–3.5), urban residents (OR = 2.0; 95% CI: 1.3–3.0), participants with 2 or more chronic conditions (OR = 2.1; 95% CI: 1.4–3.1), individuals with limitations in IADL (OR = 1.7; 95% CI: 1.1–2.8), and those reporting limitations on the GALI (OR = 1.6; 95% CI: 1.1–2.5).

**Table 1.** Distribution of Health Service Needs Among the Study Population, Adults Aged 50+, SHARE Latvia, n = 647.

Variables	Unmet needs, frequency, % (n)	No unmet needs, frequency, % (n)	Total, % (n)	OR (95% CI)	*P*	aOR (95% CI)	*P*
Gender							
Woman	32.7 (133)	67.3 (274)	62.9 (407)	**1.5 (1.1-2.2)**	**.022**	**1.6 (1.04-2.4)**	**.034**
Man	24.2 (58)	75.8 (182)	37.1 (240)	1		1	
Age (years)							
65+	33.0 (131)	67.0 (266)	61.4 (397)	**1.6 (1.1-2.2)**	**.015**	1.6 (0.9-2.8)	.099
50-64	24.0 (60)	76.0 (190)	38.6 (250)	1		1	
Education							
Tertiary	36.0 (54)	64.0 (96)	23.2 (150)	**2.0 (1.3-3.2)**	**.003**	**2.1 (1.3-3.5)**	**.003**
Secondary	32.2 (88)	67.8 (185)	42.3 (273)	**1.7 (1.1-2.5)**	**.011**	**1.8 (1.2-2.8)**	**.008**
Primary	22.0 (49)	78.0 (174)	34.5 (223)	1			
Partnership status							
No partner	30.4 (79)	69.6 (181)	40.2 (260)	1.1 (0.8-1.5)	.693	0.9 (0.6-1.4)	.784
Has a partner	28.9 (112)	71.1 (275)	59.8 (387)	1		1	
Area lived							
Urban	34.3 (141)	65.7 (270)	63.6 (411)	**1.9 (1.3-2.8)**	<**.001**	**2.0 (1.3-3.0)**	**.001**
Rural	21.3 (50)	78.7 (185)	36.4 (235)	1		1	
Economic activity (before the pandemic)							
Not active	31.2 (149)	68.8 (329)	74.3 (478)	1.3 (0.9-2.0)	.167	0.8 (0.5-1.6)	.597
Active	25.5 (42)	74.5 (123)	25.7 (165)	1		1	
Financial situation (first year of the pandemic)							
Poor	31.2 (106)	68.8 (234)	54.4 (340)	1.2 (0.9-1.8)	.217	1.2 (0.8-1.9)	.372
Good	26.7 (76)	73.3 (209)	45.6 (285)	1		1	
Financial situation (second year of the pandemic)							
Poor	32.1 (105)	67.9 (222)	53.0 (327)	1.3 (0.9-1.9)	.131	1.3 (0.8-2.0)	.298
Good	26.6 (77)	73.4 (213)	47.0 (290)	1		1	
Chronic conditions							
2+	37.0 (125)	63.0 (213)	52.7 (338)	**2.1 (1.5-3.1)**	<**.001**	**2.1 (1.4-3.1)**	<**.001**
<2	21.5 (65)	78.5 (238)	47.3 (303)	1		1	
ADL							
Yes	24.1 (13)	75.9 (41)	8.4 (54)	0.7 (0.4-1.4)	.357	1.0 (0.4-1.8)	.759
No	30.1 (178)	69.9 (414)	91.6 (592)	1		1	
IADL							
Yes	38.8 (50)	61.2 (79)	20.0 (129)	**1.7 (1.1-2.5)**	**.011**	**1.7 (1.1-2.8)**	**.020**
No	27.3 (141)	72.7 (376)	80.0 (517)	1		1	
GALI							
Limited	32.8 (133)	67.2 (272)	62.6 (405)	**1.6 (1.1-2.2)**	**0.017**	**1.6 (1.1-2.4)**	**.029**
Not limited	24.0 (58)	76.0 (184)	37.4 (242)	1		1	

*Note.* *P*-value obtained using the chi-squared test. Values in bold indicate statistical significance (*p* < .05). Adjusted for gender, age, education, family status, area lived, economic activity and economic status. The sum of n for a specific variable may be smaller than the total n due to missing data.

N = number; OR = odds ratio; CI = confidence interval; aOR = adjusted odds ratio.

As shown in Table 2, a gender-stratified multivariate analysis was conducted to understand better the determinants of unmet health services needs among women and men, adjusting for age, education, partnership status, area of residence, economic activity, and financial status. The results of the adjusted model revealed significantly higher odds of experiencing unmet healthcare needs among urban residents (OR = 1.9; 95% CI: 1.2–3.2 for women, and OR = 2.2; 95% CI: 1.1–4.7 for men), men with secondary and tertiary education (OR = 2.8; 95% CI: 1.2–6.7 and OR = 3.3; 95% CI: 1.3–8.3, respectively), women with 2 or more chronic conditions (OR = 2.1; 95% CI: 2.1–3.6) and reporting limitations in IADL or GALI (OR = 2.2; 95% CI: 1.2–3.9 and OR = 2.1; 95% CI: 1.2–3.9, respectively).

**Table 2.** Gender Stratified Unmet Health Services Needs and Associated Factors, Adults Aged 50+, SHARE Latvia, n = 191.

Variables	Woman				Man			
	Unadjusted model		Adjusted model		Unadjusted model		Adjusted model	
	OR (95% CI)	*P*	OR (95% CI)	*P*	OR (95% CI)	*P*	OR (95% CI)	*P*
Age (years)								
65+	1.3 (0.8-2.1)	.227	1.2 (0.6-2.3)	.666	**1.9 (1.0-3.5)**	**.041**	2.4 (0.8-6.9)	.107
50-64	1		1		1		1	
Education								
Tertiary	1.6 (0.9-2.8)	.120	1.7 (0.9-3.2)	.087	**3.5 (1.5-8.0)**	**.003**	**3.3 (1.3-8.3)**	**.011**
Secondary	1.5 (0.9-2.4)	.108	1.5 (0.9-2.5)	.125	**2.3 (1.1-5.1)**	**.033**	**2.8 (1.2-6.7)**	**.019**
Primary	1		1		1		1	
Partnership status								
No partner	1.0 (0.7-1.6)	.872	1.0 (0.6-1.6)	.956	0.6 (0.2-1.4)	.216	0.8 (0.3-2.0)	.574
Has a partner	1		1		1		1	
Area lived								
Urban	**1.9 (1.2-3.0)**	**.006**	**1.9 (1.2-3.2)**	**.009**	**1.9 (1.01-3.7)**	**.047**	**2.2 (1.1-4.7)**	**.030**
Rural	1		1		1		1	
Economic activity (before pandemics)								
Not active	1.5 (0.9-2.4)	.159	1.2 (0.5-2.6)	.666	1.1 (0.6-2.0)	.88	0.5 (0.2-1.5)	.230
Active	1		1		1		1	
Financial situation (first year of the pandemic)								
Poor	**1.6 (1.1-2.5)**	**.031**	1.4 (0.8-2.4)	.185	0.7 (0.4-1.3)	.292	0.9 (0.4-2.0)	.784
Good	1		1		1		1	
Financial situation (second year of the pandemic)								
Poor	**1.6 (1.1-2.5)**	**.023**	1.4 (0.8-2.3)	.251	0.8 (0.4-1.5)	.467	1.1 (0.5-2.4)	.868
Good	1		1		1		1	
Chronic conditions								
2+	**2.4 (1.6-3.8)**	<**.001**	**2.6 (1.5-4.3)**	<**.001**	1.5 (0.8-2.8)	.158	1.2 (0.6-2.4)	.539
<2	1		1		1		1	
ADL								
Yes	0.9 (0.4-1.8)	.689	1.0 (0.5-2.3)	.951	0.4 (0.1-1.8)	.227	0.7 (0.1-3.5)	.652
No	1		1		1		1	
IADL								
Yes	**2.1 (1.3-3.4)**	**.002**	**2.2 (1.3-3.8)**	**.004**	0.8 (0.3-1.8)	.525	0.8 (0.3-2.4)	.718
No	1		1		1			
GALI								
Limited	**2.3 (1.5-3.7)**	<**.001**	**2.6 (1.5-4.4)**	<**.001**	0.7 (0.4-1.3)	.310	0.7 (0.4-1.5)	.379
Not limited	1		1		1		1	

*Note.* *P*-value obtained using the chi-squared test.Values in bold indicate statistical significance (*p* < .05). Adjusted for age, education, family status, area lived, economic activity and economic status.

N = number; OR = odds ratio; CI = confidence interval.

## Discussion

Latvia has one of the highest out-of-pocket healthcare expenditures among Organisation for Economic Cooperation and Development countries^14^ and one of the highest proportions of households that experienced catastrophic health spending among the EU countries,^23^ placing a significant burden on individuals. Additionally, it had one of the highest combined mortality rates for preventable and treatable causes in the EU, nearly twice the EU average in 2020.^15^ This underscores the critical role of access to and continuity of healthcare, particularly for the elderly, who are more vulnerable to health risks and often face greater barriers to care. To the best of our knowledge, this is the first study to examine unmet health service needs among the older population in Latvia, covering both the first and second years of the pandemic.

Using data from the SHARE study, we found that unmet health service needs among individuals aged 50 years and older during the COVID-19 pandemic in 2020 and 2021 were frequently reported. In Latvia, 29.5% of individuals were unable to access at least some form of healthcare services during this period. Our findings align with epidemiological trends observed during the pandemic, positioning our results towards the upper range of prevalence reported in comparable studies. For instance, in Canada, during the autumn of 2020, 25% of participants reported difficulties accessing healthcare services.^24^ Similarly, a systematic review published in 2021 found that global healthcare utilisation decreased by approximately one-third.^25^ A study encompassing the first year of the pandemic revealed significant variation in unmet health service needs among individuals aged 50 and older across the EU, ranging from over 35% to as low as 7%.^10^

### Sociodemographic Factors

Studies indicate that unmet health service needs during pandemics were lower among the elderly,^8,24^ with some suggestions that older age may have had a protective effect against foregoing, postponing, or being denied healthcare during the outbreak, possibly due to continued support from others and a lower reluctance to seek medical care.^11^ Our study first observed higher unadjusted odds of unmet health service needs among individuals aged 65 and older than those aged 50 to 64. However, this association diminished after adjusting for sociodemographic factors, suggesting that these factors play a critical role in explaining age-related differences in unmet health service needs during pandemics in Latvia, as discussed further.

The observed higher odds of unmet health service needs among urban residents in this study align with earlier research on the 50+ population in Europe, which has shown that limited healthcare access was more prevalent among individuals living in urban areas during the COVID-19 outbreak.^11^ Before the outbreak, urban areas were considered facilitators of healthcare access due to the higher concentration of primary care providers, specialists, and hospitals.^26^ Furthermore, urban-rural differences in healthcare access have been linked to the impact of urban living on the likelihood of encountering unmet health service needs due to financial constraints.^27^

In our study, 32.7% of women and 24.2% of men reported unmet health service needs during the 2 years of the COVID-19 pandemic in Latvia (2020 and 2021). The ratio of gender differences is similarly reported in the study conducted on older populations across Europe during the pandemic, showing comparable disparities in unmet health service needs.^8^ Brown et al^28^ found that women were less likely than men to proceed with surgeries associated with a high risk of COVID-19 infection and mortality. Gender disparities in healthcare utilisation and outcomes have been documented in previous studies before the pandemic. Women tend to live longer than men and often face prolonged periods of activity limitations.^29^ Furthermore, Guessous et al^30^ found that women were more likely than men to forgo healthcare for economic reasons. This highlights the persistent gender disparities in meeting healthcare needs among the older population, which the pandemic may have further intensified. Thus, gender-stratified analyses were further made in our study. The insufficient utilisation of healthcare services by older women during pandemics in relation to their actual needs can deepen health inequalities, increase dependence on caregivers and others, and place additional pressure on financial resources in future.

An initial association was observed between education level and unmet health service needs in our study. However, after adjustment, it remained significant only among men, with those having higher education levels showing higher odds of unmet health service needs. Previous research has shown that men are more likely to delay seeking healthcare services and to perceive care as non-essential.^31,32^ Furthermore, the relationship between education and unmet health service needs is complex. On the one hand, evidence suggests that respondents with higher education levels were more likely to forgo or postpone healthcare, possibly because they had more frequent healthcare utilisation before the pandemic.^33^ On the other hand, individuals with higher levels of education were more likely to perceive barriers to accessing healthcare, particularly due to epidemic control measures.^11^ Together, these factors may explain the higher rates of missed healthcare, as men with higher education levels might have been more willing to accept the postponement of their medical appointments.

Lower income and lower levels of employment have been identified as predictors of unmet health service needs in research conducted before the pandemic.^30,32^ Similarly, Anderson et al^34^ found increased rates of forgone medical services among individuals with lower household incomes or those who were unemployed in a sample from the United States during the initial phase of the COVID-19 pandemic. It has been suggested that the increased risk of forgoing care among the economically vulnerable may be related to their financial and general insecurity, which may have increased during the pandemic crisis and may have stressed their general feeling of insecurity.^12^ Our study initially identified a positive association between unmet healthcare needs during the first and second years of the pandemic among women in the gender-stratified analysis. However, this association diminished after adjusting for sociodemographic factors, suggesting that other variables—such as place of residence—play a more significant role in explaining differences in unmet healthcare needs during the pandemic in Latvia, as previously discussed.

### Chronic Conditions and Limitations

It has been suggested that the COVID-19 pandemic has worsened the condition of non-communicable disease patients, thereby posing a greater threat to the sustainability of healthcare systems.^13^ Furthermore, several studies have reported that delays in referrals caused by the COVID-19 pandemic increased mortality among patients with chronic conditions.^35,36^ A study analysing access to healthcare services in Canada during the pandemic found that participants with chronic conditions were more likely to report difficulties accessing services and avoid hospitals or doctors when needed.^24^ Additionally, a study covering adults aged 50 years and older in Europe suggested that the healthcare demand during the pandemic was correlated with initial healthcare habits for a given health status, which reflects preferences regarding health and healthcare and initial difficulties in accessing health service.^12^ Our study identified a significant association between unmet health service needs and having 2 or more chronic conditions. However, this association remained significant only for women in the adjusted gender-stratified analysis. This finding highlights the need for greater attention to individuals, specifically women, with chronic conditions in future healthcare planning, prioritising their care both during emergencies and in the long term.

The results of our study revealed that functional limitations were significant predictors of difficulties in accessing healthcare among individuals aged 50 and older during the COVID-19 pandemic in Latvia. This finding aligns with earlier studies, which demonstrated that older adults with dependencies in limitation faced a higher risk of unmet health service needs.^32^ Previous research has consistently shown that greater mobility limitations are strongly associated with unmet health service needs and poorer health outcomes,^37,38^ underscoring the importance of considering functional limitations when identifying individuals at risk of requiring healthcare support.^31^ In our study, the adjusted odds of unmet health service needs were significantly higher for women reporting limitations in IADL and GALI.

Even before the pandemic, the urgent need to address an ageing population’s unmet care and support needs has been addressed by designing services and solutions that align with what older people need or want.^39^ During the pandemic, many public services—including healthcare—shifted to digital platforms.^5^ An analysis of the impact of COVID-19 on the Latvian population aged 50 and older identified inequalities in digital skills and access to technology, highlighting the need to strengthen digital competencies among older adults, as these skills are increasingly important for meeting basic needs and maintaining health. The shift to digital platforms disproportionately affected this age group, which often faces barriers such as limited digital literacy, restricted access to technology, and related challenges.^40^ Thus, enhancing digital literacy among the elderly could help mitigate these barriers and reduce disparities in access to healthcare.

Findings from this study highlight systemic weaknesses in ensuring continuity of care for high-risk groups. Enhancing healthcare system resilience involves ensuring that service delivery remains uninterrupted, with particular attention to protecting access for those most vulnerable. This includes implementing flexible care models that integrate both in-person and remote consultations into regular practice, as well as investing in equitable digital infrastructure across regions. Furthermore, strengthening cross-sectoral coordination and maintaining access to essential primary and chronic care are vital towards improving the care that meets the specific needs of older adults.

## Conclusions

In conclusion, this study offers novel insights into the unmet health service needs of the older population in Latvia during the first 2 years of the COVID-19 pandemic, highlighting significant disparities in Latvia. Gender, socioeconomic factors such as urban residence and higher education, chronic conditions, and functional limitations were substantial determinants of unmet healthcare needs among the older population. The most affected groups included urban residents, older men with higher education and older women with multiple chronic conditions and functional impairments. These findings underscore the need for targeted interventions to reduce access barriers and address healthcare inequalities during public health emergencies.

While the observed patterns are broadly consistent with trends across EU countries, Latvia faces unique systemic challenges, including limited healthcare funding, high out-of-pocket payments, and uneven service availability. Therefore, comprehensive and locally adapted strategies are essential to ensure equitable healthcare access in both crisis and routine care.

## Study Limitations

We acknowledge several limitations of this study. First, our analyses relied on self-reported data, which may introduce subjective interpretation from respondents. At the same time, self-reported data provides valuable insight into individuals’ perceptions and experiences, and the SHARE questionnaire follows the ex-ante harmonisation approach for its design, thus being a reliable source of self-reported information.

Questions that captured the unmet health service needs among older adults in the context of the COVID-19 pandemic in the SHARE questionnaire are quite specific, focussing on fear of infection, appointment postponement by healthcare providers, and difficulty in scheduling appointments. As the questions were explicitly framed around the COVID-19 outbreak period, they may not capture ongoing or chronic unmet healthcare needs unrelated to the pandemic, nor do they address financial barriers, transportation challenges, lack of insurance, or systemic issues in healthcare access. No information was available on the nature or severity of medical problems in cases where curative care was sought, nor on whether lockdown measures or healthcare system prioritisation policies influenced access. The questions do not assess the severity of the unmet need or the health consequences of forgoing or postponing care. Some unmet needs may be minor, while others could have profound health implications; however, this distinction is not captured.

Another limitation of this study is the reduced sample size resulting from disruptions to SHARE data collection caused by the COVID-19 pandemic, which may have influenced both the size and composition of the sample reached. Nonetheless, to the best of our knowledge, this represents the first attempt to analyse unmet health service needs during the pandemic within this defined target group in Latvia. Future studies could benefit from targeted primary data collection with predefined power calculations to confirm and build upon the current findings.

Altogether, this highlights the importance of future research, including health-related data and healthcare system records, to better understand the interaction between medical necessity, policy restrictions, and healthcare accessibility during crises.

## Supplemental Material

sj-doc-1-inq-10.1177_00469580251371425 – Supplemental material for Inequities in Health Service Needs Among Older Adults During COVID-19: A Gender-Stratified Analysis Using Longitudinal SHARE Data Supplemental material, sj-doc-1-inq-10.1177_00469580251371425 for Inequities in Health Service Needs Among Older Adults During COVID-19: A Gender-Stratified Analysis Using Longitudinal SHARE Data by Aija Bukova-Žideļūna, Anda Ķīvīte-Urtāne, Lauma Sprinģe, Ilze Priedīte, Diāna Baltmane and Šime Smolič in INQUIRY: The Journal of Health Care Organization, Provision, and Financing
